# Migrating from user fees to social health insurance: exploring the prospects and challenges for hospital management

**DOI:** 10.1186/1472-6963-12-174

**Published:** 2012-06-22

**Authors:** Roger A Atinga, Sylvester A Mensah, Francis Asenso-Boadi, Francis-Xavier Andoh Adjei

**Affiliations:** 1 Department of Public Administration and Health Services Management, University of Ghana Business School, Legon, Accra, Ghana; 2 National Health Insurance Authority, Accra, Ghana

## Abstract

**Background:**

In 2003 Ghana introduced a social health insurance scheme which resulted in the separation of purchasing of health services by the health insurance authority on the one hand and the provision of health services by hospitals at the other side of the spectrum. This separation has a lot of implications for managing accredited hospitals. This paper examines whether decoupling purchasing and service provision translate into opportunities or challenges in the management of accredited hospitals.

**Methods:**

A qualitative exploratory study of 15 accredited district hospitals were selected from five of Ghana’s ten administrative regions for the study. A semi-structured interview guide was designed to solicit information from key informants, Health Service Administrators, Pharmacists, Accountants and Scheme Managers of the hospitals studied. Data was analysed thematically.

**Results:**

The results showed that under the health insurance scheme, hospitals are better-off in terms of cash flow and adequate stock levels of drugs. Adequate stock of non-drugs under the scheme was reportedly intermittent. The major challenges confronting the hospitals were identified as weak purchasing power due to low tariffs, non computerisation of claims processing, unpredictable payment pattern, poor gate-keeping systems, lack of logistics and other new and emerging challenges relating to moral hazards and the use of false identity cards under pretence for medical care.

**Conclusion:**

Study’s findings have a lot of policy implications for proper management of hospitals. The findings suggest rationalisation of the current tariff structure, the application of contract based payment system to inject efficiency into hospitals management and piloting facility based vetting systems to offset vetting loads of the insurance authority. Proper gate-keeping mechanisms are also needed to curtail the phenomenon of moral hazard and false documentation.

## Background

There has been growing interest for the implementation of social health insurance in most developing countries fuelled in part by the negative repercussion of user fees on utilisation rates and healthcare management. Compared to social health insurance, user fees subject hospitals to nominal provision of essential services [1]. Against a background of inadequate budgetary support, user fees expose hospitals to critical logistical and medical supply shortages, dysfunctional medical equipment, weak management systems and poor health professional motivation [2,3]. These problems get complicated by low unit cost of service charges, the burden of new and emerging diseases and the fact that hospitals consume excessive resources in relation to the services they produce [4-7]. Additionally, rising expectations as clients become more and more critical about service quality, inadequate staffing levels and poor infrastructure all combine to pull strings to hospital management. In some instances, the inability of government to identify and cope with the underlying problems that operate to produce constraints to management of hospitals have usually forced healthcare managers into frustration.

In light of these problems, it is argued that user fee offer no practical incentives for improving healthcare management unless health insurance is introduced [3]. Most governments are therefore embarking on a transition to social health insurance as a strategy to improve efficiency and cost recovery levels of hospitals. Social health insurance has the potential of increasing hospitals economies of scale to cope with service delivery. Thus, in 2005 the government of Ghana introduced a National Health Insurance Scheme (NHIS) to address the many problems that the health sector inherited from the cash and carry system. The emergence of the NHIS created an internal market arrangement within which service provision and purchasing of health services are provided under different platforms. The National Health Insurance Authority (NHIA) purchases health services for policy holders while healthcare institutions such as hospitals, clinics and health centres engage in service provision.

**Figure 1:**
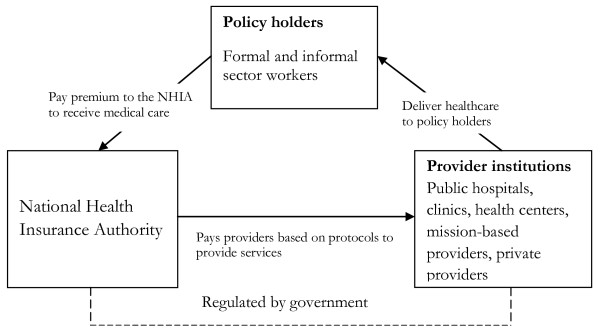
Relationship between actors of the NHIS.

In principle, the NHIA handles relationship with the public and it is only when a registered member of the scheme becomes a patient that contact with the provider is initiated. The NHIA represents the public and ensures that they receive the required benefit packages of health services specified in the insurance protocol. The Authority maps out pressing health needs of the population and pays provider institutions to provide healthcare to registered members. The relationship between policy holders, the NHIA and provider institutions is illustrated in Figure 1. This study focuses on the relationship between the NHIA and provider institutions to determine whether the later profits or encounter problems arising out of such a relationship.

Although policy makers have not clearly spelt out guidelines against which the purchasing body and providers can operate in this context, the potential advantages cannot be blurred in obscurity. An important component of this restructuring model is its capacity to improve managerial effectiveness [8] since it enables providers to fully concentrate on healthcare delivery without being overburdened with the task of estimating the cost of providing medical care to health consumers. Another incentive of this system is its ability to empower the purchasing body and by implication, the NHIA to use its purchasing power to compel service providers to offer quality care. It is further argued that the purchasing authority is well placed to match services with need and to alter the mix of services available by acting in the interest of the population of policy holders. Also associated with the model is the efficiency gains and accountability in providing services using service level arrangements [9].

Despite the incentives of this arrangement, the threats of splitting purchasing and service provision cannot be discounted. Key among them is the tendency of late reimbursement to provider institutions which can culminate in financial volatility and distortion of their budgetary plans. Providers can also operate with constraints when the purchaser pays limited attention to prices and other market signals. Under such circumstance, provider institutions may be compelled to compromise service quality over cost [10]. Finally, the separate streams of managing financial resources on the one hand and providing health services on the other may result in the provision of questionable quality when providers feel they are functionally losing from the arrangement.

The traditional sources of funds to hospitals in Ghana include health insurance, internally generated funds or user fees, central government allocations and donor aid [11]. Health insurance and user fees are the most common cost recovery strategies of hospitals however. More than three-quarters of the funds accruing to hospitals are from the national health insurance. Provider payment mechanism follows protocols established by the NHIA. Health providers submit bills on monthly basis to the NHIA for reimbursement. However, the Authority reimburses only bills certified to be authentic (free from errors, falsification and misquoting of the Ghana DRG system). Reimbursements are usually made either in full or instalment. The option for a particular method of payment depends on the outcome when the aggregate cost of payment and number of health facilities to be reimbursed are entered into the equation. Motivated by the classical agency theory which holds onto competitive contracts for specific services [12] the NHIA is currently piloting a capitation payment system to improve pricing and reimbursement activities as well as technical and allocative efficiency of hospitals.

Regardless of the payment method applied, the fact remains that activities of the purchasing body (NHIA) has trickledown effect on provider institutions that are at the receiving end. This study makes no pretence at examining hospital performance under the scheme nor draw best practices of hospital management under health financing reforms in other jurisdictions. Rather the study seeks to examine whether separation of purchasing and service provision translate into challenges or opportunities in the management of accredited hospitals. By so doing, the study departs from previous researches in the literature that examined sustainability and impact of the scheme on stakeholders and financial performance of the mutual health insurance schemes [13,14].

## Methods

Data for the study was collected from five administrative regions in Ghana (Greater Accra, Brong Ahafo, Ashanti, Eastern and Volta). The selection of these regions was motivated by a similar research conducted by the first Author and his colleagues in the three northern regions, central and western regions [15]. Finding of the previous research suggested an expansion of the regions to better inform policy Action.

In each selected region of the study, a sample of three districts and the respective public district hospitals that served as referrals facilities to all the clinics and health centres in the catchment area were selected. The full complement of a district and its hospital was selected by applying two criteria: in the first place, the district must have a well functioning mutual health insurance scheme. Secondly, the district public hospital must have been an accredited provider for at least five years. The intention was to select only hospitals with fair experience of service provision under the scheme. Prior to the selection of the specific hospitals, we discovered that many of them were qualified to be selected per the criteria outlined above. We however selected the hospitals that were geographically accessible.

In each hospital, key informants (Health Service Administrators, Heads of pharmaceutical services, Chief Accountants and hospital-based Scheme Managers) were purposely chosen [16,17] to reflect their role within the management structures of hospital in Ghana which inter alia include, decision making, planning, directing and resource allocation. Being at the helm of management, they were more inclined to identify the benefits and problems confronting hospitals that depend on subsidies from the NHIA. The initial intention was to interview the different mix of key informants outlined above in each hospital. But this effort was masked by the absence of pharmacists with a minimum of two years work experience in five of the hospitals. Further, in many of the hospitals, the claims managers were very new at post and under temporal engagement with the hospitals. They were therefore excluded from the sample.

To elicit detail and unrestricted responses that reflected the underlying objective of the study, personal interviews were preferred for two reasons. Firstly the acute shortage of health professionals in district hospitals tended to generate heavy workload and busy schedules for managers to the extent that it was highly impossible to bring them together for a group discussion. Secondly, personal interviews compare favourably with focus group discussion because as indicated by De Allegri et al. [18] they offer individuals the opportunity to freely articulate their opinions without fear of being criticised by others.

At the preliminary stage, the instrument developed for the study was piloted in two district hospitals in the Eastern and Greater Accra regions. The aim was to detect inconsistencies and to determine the strength and validity of the themes raised. Findings of the pilot study provided significant information for the development of the final instrument. During interview with respondents in the pilot study, we found that some of the themes could be better understood and well interpreted when they are structured into closed-ended questions. We were guided by this and therefore introduced a likert scale to some of the questions. The actual study was conducted using a semi-structured interview guide that captured broad themes relating to cash flow and reimbursement pattern; claims management; level of improvement of capital equipment (e.g. vehicles, medical equipment etc); logistics; human resource; drugs and non drug consumables. Respondents were however given enough degree of flexibility in responding to the themes raised. The reason was to offer participants the opportunity to ask questions and bring out other relevant suggestions to be incorporated into the study using a continuous validation procedure [19]. This approach eventually proved helpful. For instance, the informants identified and incorporated medication issues, workload analysis, waiting hours and deficient networking systems into the objective function. These emerging themes were analysed together with those specified in the interview guide. We were being mindful of the fact that closed ended questions that reflected level of improvement of key performance areas of the hospitals could be given different interpretations by the mix of respondents in the sample. In this light, only the Health Service Administrators of the various hospitals were allowed to respond to such questions.

Given that three of the Authors are NHIA employees with positions and opinions on the issue under investigation, a possibility of bias and conflict of interest could have surfaced when they were allowed to participate in the data collection process. To give the study its original meaning therefore, the first Author led a team of four research assistants (students pursuing Master degrees in various disciplines) to collect the data. A two day training programme was organised for the research assistants during which they were schooled on approaches to qualitative data collection; how to conduct oral interviews; approach the managers and the methods of taking detail field notes.

Ethical approval to conduct the study was sought from the health directorates of the various regions. They were contacted by mails that explained the study’s purpose and the research team involved. All the Managers contacted subsequently endorsed the study as one that would shape policy direction. Further approval was sought from the health services administrators and medical superintendents of each hospital. Obtaining explicit consent from people in authority such as these managers of the hospitals aided the researchers in obtaining maximum cooperation from all the participants.

Data was analysed under common themes [19,20]. Themes extracted during interview with the key informants were categorised under a number of broad headings. Notes taken from the interviews were typewritten by the first author together with the research assistants. The typed notes together with the completed questionnaires were then handed down to the last author to present the results though with minimal involvement of the first author whose role was limited to making clarifications. Illustrations of thematic areas are represented by verbatim quotations from the informants. Structured closed-ended themes were analysed using descriptive statistics with the aid of SPSS version 18.

## Results

A total of 44 key informants participated in the study of which 15 were Health Service Administrators. Additionally, the study attracted 10 Pharmacists, 14 senior accountants and 5 scheme managers of the hospitals (Table 1). Average hospital tenure (AHT) of the informants shows that the Health Service Administrators spent the most years working in the hospitals (AHT = 4.8). This is followed in order by the Accountants (AHT = 3.5), Pharmacists (AHT = 2.8) and the facility-based claims managers (AHT = 2.0). The distribution of job tenure suggest the informants have had fair experience on the job to enable them provide credible information about successes and problems of hospitals under the scheme.

**Table 1:** Characteristics of key informants

**Key informants n = 44**	**n**	%	**Average hospital tenure (years)**
Health Service Administrators	15	34.1	4.8
Head Pharmacists	10	22.7	2.8
Accountants	14	31.8	3.5
Claims managers	5	11.4	2.0

### Claims processing and management

A major problem that characterise the implementation of the health insurance scheme is technological setback. Effective management of claims still remain a difficult task for many hospitals. Results of the study show that all the hospitals still rely heavily on the manual method for processing claims. This manual system is not only prone to errors, but also causes undue delays in processing claims. Processing of claims in hospitals with even lower utilisation rate tend to take several weeks. The average minimum number of weeks for claims processing in the hospitals was 2 while the maximum is 4 but it can be more for hospitals with higher utilisation. Although delay in processing of claims was attributed largely to the use of the manual system as opposed to the application of a more robust computerised system, several other factors were identified as obstacles to fast-tracking claims processing.

"“A lot of paper work characterized by inputting of large volumes of client data”."

"“Limited number of staff with sufficient knowledge in claims management”."

"“Poorly written patient data. Health professionals do not often take their time to clearly and neatly write down the disease history and treatment procedure. Thus claims processors often have to spend a great deal of time making meaning of what is written”."

"“Increased utilisation generating heavy workload”."

As a mechanism of cost containment, the NHIA has adopted a number of approaches including the use of clinical audits and stringent claims vetting procedure to deal with fraudulent bills. Several accredited hospitals have come under various sanctions by the NHIA including rejection of claims on the basis of falsifications. Whether this reflects lack of providers understanding of the insurance protocols or the difficulty of interpretation of the Ghana DRG-based reimbursement system remains unclear. It was however pointed out that claims submitted for reimbursement could be rejected or reduced due to omissions and falsifications. Other factors according to the informants are:

"“Errors pertaining to wrong diagnosis and prescriptions”."

"“Overbilling of drugs and services provided”."

"“Misappropriations of drugs and services provided”."

"“Misappropriation of the established tariff structure”."

"“Misquoting of the Ghana DRG tariffs: Not in line with what has been established by the NHIA”."

"“Treatment of patients whose ID cards are expired”."

"“Non adherence to laid down protocols”."

### Cash flow and reimbursement of providers

**Figure 2:**
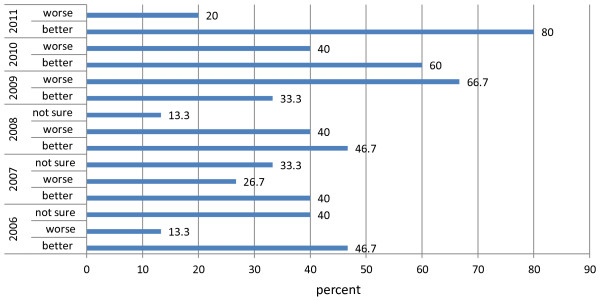
Perception of yearly improvement of reimbursement (2006–2011).

Financing healthcare delivery has always been a problem in most developing countries. Even where social health insurance schemes are established, it may still be problematic when reserves of insurance agents offer limited capacity to engage in prompt reimbursement. Between 2006 and 2009, a larger number (40%) of the hospitals were reimbursed within 3 months. 13.3% reported receiving reimbursement after 4 months and 46.7% of the hospitals were reimbursed between 4 and 6 months. Generally the informants were of the view that reimbursement was worse and unpredictable in 2009 as it took the health facilities an average of 4 months to be reimbursed. However, there is some level of optimism in recent times. The opinion formed was that reimbursement is better especially within the last two years (Figure 2). 60% of the informants were of the view that reimbursement in 2010 was better compared to 40% who reported that reimbursement was worse. A remarkable improvement of reimbursement occurred in 2011. An overwhelming number (80%) of the informants held that reimbursement is getting better compared to 20% who held contrary opinions (Figure 2). This might be attributed to the recent moderate claims payment reforms initiated by the NHIA to ensure that every accredited provider is reimbursed within 4 weeks after submitting claims. Although repayment is getting better, in principle providers are still not reimbursed within the four weeks time line as specified in the NHIS Act (2003, Act 650).

Nonetheless, reimbursement may be irregular but it still serves as a big incentive and opportunity for hospitals. Many of the health facilities experienced increased revenue under the current payment system. 47% of the informants indicated that revenue generation and cash flow of the hospitals is very high. A further 27% reported revenue increase under the scheme (Table 2). As pointed out by an informant, “the hospital’s revenue has increased substantially due to improvement of reimbursement”. Another informant remarked “unlike the first three years following the implementation of the scheme, reimbursement is getting better and inflow of funds has improved”. A cross section of the informants commented positively on revenue generation under the scheme.

**Table 2:** Level of improvement in key performance areas of the hospitals

**Item**	**Very high**	**High**	**Neither high nor low**	**Low**	**Very low**
*n = 15	%	%	%	%	%
Improvement of drug procurement under the NHIS	33.3	33.3	26.7	6.7	-
Drug availability	20.0	40.0	20.0	20.0	-
Availability of basic non-drug consumables	40.0	26.7	26.7	6.6	-
Revenue generation and cash flow	26.7	46.6	6.7	20.0	-
Staffing (especially hiring of casual staff)	6.7	60.0	26.7	6.6	-
Facility maintenance	33.3	20.0	26.7	20.0	-
Purchase of medical equipment	6.7	33.3	33.3	26.7	-
Acquisition of vehicles	-	13.3	26.7	46.7	13.3
Training programmes	13.3	46.7	26.7	13.3	-
Other investment	6.7	20.0	33.3	26.7	13.3

*Opinions of the 15 health service administrators of the hospitals.

"“The NHIS has boosted revenue of the hospital. What I can say is that revenue generation has improved”."

"“The hospital’s revenue is good and consistent”."

"“Monies come in bulk so it becomes relatively easy to do effective planning”"

"“Despite the fact that the current tariff structure is low, cash flow is still good”."

Some of the informants also perceived that the current tariff structure creates cash flow problems. The reasons advanced were straightforward.

"“The current tariff structure is low making cost recovery of health delivery difficult”."

"“We cannot pretend that all is well when it is not. We are indebted to some suppliers because we are not paid on time. They are on us every day but we keep telling them we are not paid yet. So it is difficult to claim that revenue is high when in fact we are indebted to our suppliers”."

"“The issue is about the current tariff structure. It is too low and costs of supplies are increasing. Our budgets are being over stretched. To be realistic, how can we rely on the low tariffs to effectively provide care for registered members and the larger numbers of exempt group?”"

The argument is that hospitals expenditures are soaring but this has not been corroborated with an upward review of the tariff structure established in 2008. Although a genuine concern, it is important to state that social health insurances are by nature driven by higher expenditures. Transaction cost relating to procurement and supplies could even be more when there is excessive consumption. Whatever the situation, the pattern of response suggests that the hospitals are better-off under the health insurance regime. What is needed is efficient management of financial resources to minimise wastages.

### Stock levels of drug and non-drugs

Stock levels of both drug and non drug consumables in the health facilities attracted divergent views. While a number of the informants felt that the scheme has marginally contributed to improvement of stock levels of drugs, others thought the scheme has greatly enhanced hospitals capacity to procure large quantities of drugs.

"“The NHIS has enhanced the capacity to procure more drugs. We now visit the Regional Medical Stores (RMS) more often hence drugs are readily available”."

"“The hospital is not able to have in stock sufficient drugs due to increased utilisation of health services”."

"“Stockout occur occasionally because the hospital hardly runs out of drugs except when the hospital faces extreme pressure from patients”."

"“Availability of drugs under the scheme is good”."

"“The scheme has improved upon drug availability. This is because the hospital is able to purchase drugs from the open market”."

Findings of the study generally indicate that the hospitals do not face chronic shortage of drugs. As reported in Table 2 many (40%) of the informants maintained that drug availability under the scheme is high. It was however observed that certain essential but expensive drugs covered by the scheme were not readily available in the hospitals dispensaries. Thus patients were compelled to make upfront payment for such drugs in the pharmaceutical stores. The hospitals also faced challenges relating to maintaining adequate stock level of non-drugs such as infusions, cotton, gloves, gauze, needles and syringes, catheter, cord clamp, etc. A major concern was that the current tariff structure is a constraint to maintaining appreciable stocks of non-drugs. The informants had mixed opinions regarding the impact of the scheme on procurement of non-drugs.

"“Non-drugs are not always available as expected due to the current low tariff structure”."

"“Non-drugs cannot always be adequately procured to meet increased utilisation”."

"“Availability of non-drug consumables has improved”."

"“Because revenue has improved it is easy to procure non-drugs in large quantities to cope with the larger number of patients reporting for medical care”."

"“There is moderate availability of non-drug consumables. It can be improved when the current tariff structure is reviewed upwards”."

In spite of the disparaging opinions, results in Table 2 manifest that on the average stock of basic non-drug consumables was rated by the informants to be high.

### Logistics and human resources

An inherent problem of user fees is that it threatens the potentials of health providers to make significant investments. The reason is that under user fee systems, health facilities rely very much on government subventions to provide healthcare and even procure logistics [21]. Social health insurance however creates some level of autonomy for hospitals to manage their own resources. This being the case, we sought to find out whether under the health insurance scheme, hospitals are able to invest in capital equipment, undertake effective maintenance of facilities and capital equipment and engage the services of more casual and support staff to complement the existing limited number of health professionals. 46.7% of the informants were of the view that acquisition of vehicles such as ambulances and others to improve service delivery is low. 33.3% reported high purchase of medical equipment under the scheme (Table 2). This does not presuppose that such hospitals were in a position to acquire sophisticated medical equipment to deal with all reported cases. Rather they were able to procure medical equipment that can enable them deliver primary care to meet local needs. It was found that when the hospitals receive reimbursement, much of it is allocated to priority areas such as printing of patient folders, outpatient and inpatient care, supplies and deficit payment to suppliers. Hence there is so much stress on their budgets and that makes them incapable of purchasing vehicles.

Investment in human resources has generally improved. 60% of the informants reported increased hiring of casual staff to reduce the work load of clinical staff (Table 2). Each of the hospitals recruited at least 12 casuals to assist in various clinical functions as well as errands. One of the informants commented “we don’t have to allow senior nurses to send junior ones who are equally busy to pick just a folder”. That certainly will delay treatment procedures. We have addressed this by engaging the services of casuals staff to assist our medical staff”.

### Other managerial implications of the scheme

The introduction of the NHIS was fuelled in part by the need to remove financial barriers of access to healthcare and to enable health providers eliminate administrative and clinical bottlenecks that hitherto posed problems to service quality delivery. It is more than half a decade into the operation of the scheme and the very problems that necessitated the establishment of the scheme somewhat persist. Key among these challenges are high workload generated by increased utilisation, lack of logistics, inadequate stocking of medication and networking (Table 3). Apart from these challenges, long waiting hours also emerged as a critical problem. As service utilisation increases, an inherent problem of the hospitals is how to cope with long patient waiting hours. In particular, hospitals with large OPD attendance and limited medical personnel struggled to deliver care within reasonable timeline. In such hospitals, the average waiting hours could be more than three hours. The difficulty of managing long waiting hours was common to many of the health facilities.

**Table 3:** Summary comments of other challenges facing the hospitals

**Operational challenge**	**Description**
High workload	- One of the major concerns was that the increased utilisation has put so much pressure on the limited medical staff in the facilities. There is large health worker-patient ratio and this affects the provision of responsive care.
	- Inadequate numbers of health staff was mentioned as a key cause of long waiting hours.
	- The absence of people with professional knowledge of claims processing and management.
Logistics/equipment	- Lack of ambulance for referrals especially on emergency cases.
	- Lack of sophisticated surgical equipments to reduce referrals of cases to the regional hospitals.
	- Inadequate medical equipments.
	- The absence of operating theatres in the facilities to cope with new and emerging diseases.
Medication	- Many of the complaints related to the fact that the Essential Drug List (EDL) does not adequately cover a wide range of drugs. It is therefore difficult for patients to comprehend why they are not entitled to unlimited drugs under the scheme.
Networking	- Networking is a problem. As a result, some patients present expired ID cards with valid dates on them for treatment.
	- The absence of effective networking system makes activities of hospitals more difficult.
	- Because the system is not properly networked, there were instances when unregistered members scan their passport size pictures on ID cards of active members in order to receive medical care.

"“Long waiting hours is a major headache of the hospital. There is large health worker: population ratio hence it is really difficult to ensure that a patient spends little time before accessing care”."

"“Utilisation of health services has increased almost four fold in this hospital as of last year. So we encounter long waiting hours every day”."

"“People are becoming more conscious of their health so it is not surprising that people report minor illness to the hospital. Because of this, we encounter long waiting hours but that is not to say that we are not doing anything about it”."

## Discussion

One of the numerous success stories of the NHIS is the free access to healthcare (for registered members, the poor and other exempt groups) and the fact that the introduction of the scheme has led to improvement in the health seeking behaviour and choice of providers across the population of people in the country [22]. However, the scheme cannot be looked at in a vacuum; it needs to be connected to better operations of hospitals that form an integral part of the social and medical organisation of the health system [23]. Hospitals consume a disproportionate share of resources in many countries and it is even likely to be more in insurance based health systems. Yet little attention has been directed at how hospitals thrive under health insurance regimes. This study focused on the success and constraints of hospital management arising out of decoupling purchasing and provisions of health services. The study employed qualitative methods by listening to people in management positions of accredited district hospitals so as to understand the key factors of their success or otherwise [24].

Findings of the study reveal poor management of claims by the hospitals. This was attributed largely to technical deficiencies such as the application of manual systems [25]. Over reliance on such less innovative techniques was seen as a roadblock to fast tracking claims processing. Manual methods also exposed claims handling to errors. Surprising though, little or no efforts are being initiated by the hospitals to support the training of people to effectively manage claims. Many of the health facilities had only one claims processing officer, which invariably suggests that delays should not be doubted. This finding necessitates exploring the design of computerised software that allows claims to be processed very easily. Computerisation will provide partial answers to errors in claims processing and reduce variability of information flow from accredited providers to the NHIA.

Unpredictable reimbursement pattern was identified as a managerial bottleneck of the hospitals. Erratic reimbursement distorts the budgetary settings and planning of hospitals [24]. Delays in reimbursement reflect two things: delays in vetting claims on the part of the mutual health insurance schemes or cash flow problems by the health insurance authority. To minimise the duration of claims vetting, there is the need to pilot a facility-based vetting system. Under this arrangement, the health insurance authority can recruit and train people to be stationed in hospitals and tasked with vetting claims before they are submitted for reimbursement. The merit of this arrangement is that it has the potential of significantly reducing claims handling load of the insurance authority.

Reimbursement may appear erratic but the scheme has greatly improved the hospitals finances. This is comprehensible given that hospitals are better off receiving bulk sum of money during reimbursement than in bits as is the characteristic of user fees. Given that the scheme has resulted in improved cash flow, the expectation is that hospitals purchasing power should rise in the function. But this is apparently not the case, suggesting that efficiency methods are not well tapped. Therefore, hospitals could be paid a flat rate to treat all clients reporting for medical care because contracts that reimburse them for all incurred cost provide no incentive to be cost efficient and encourages them to attend to as many clients as possible [26].

Prepayment mechanisms potentially cause frequent depletion of medical supplies. Given that the number and kinds of patients utilising hospital services is partly explained by access and that prepayment mechanisms trigger increases in utilisation then frequent depletion of medical supplies should be anticipated. Stock levels of non-drugs were reported to be diminishing frequently in some of the hospitals due to high utilisation rate. Large procurement of non-drugs is peak during reimbursement but when payments are delayed stock levels decline. Hospitals inability to constantly maintain adequate stock of non-drugs was attributed to the current tariff structure which was perceived to be low in relation to current market prices of medical goods and services. This suggests an upward review of the tariff structure to boost the capacity of hospitals to procure sufficient supplies. Nonetheless, the linkage between cash flow improvement and the inability to maintain adequate stocks all year round is a complex phenomenon that demands a complex solution. An upward review of the current tariff structure is necessary but not sufficient to overcome shortage of medical supplies. Therefore an important strategy is to inject efficiency into the management structures of the hospitals. Perhaps, an optimal solution lies in prudent procurement practices.

Finding of the study suggests some level of optimism on the part of health facilities in minimising stock out rates of drugs. The general opinion formed was that the scheme has stimulated significant improvement on the availability of drugs. This may be attributed to the new drug tariff structure introduced by the NHIA, the Ghana DRG system (payment according to disease group) where drugs are billed separately on top of fixed DRGs. This tariff structure also includes some expensive drugs that were previously excluded from the essential drugs list.

The fact that moral hazard has been identified in this study as a management constraint suggests weak gate-keeping systems. Majority of the hospitals do not have appropriate systems in place to enable them detect and turn away self-referred clients who were likely to be presenting trivial conditions for treatment. This has resulted in the phenomenon of moral hazard where subscribers tend to behave in such a way as to increase the likelihood or volume of risk against which they are insured [27]. Moral hazards have undesirable consequences including frequent depletion of medical materials and long waiting hours.

## Conclusion

In healthcare context, hospitals are largely seen as targets for health financing reforms for reasons of improving access, healthcare delivery and cost recovery. More often however, evidence on the management of hospitals under health financing reforms, particularly, transition to social health insurance is on a low profile. Understanding the success and challenges of hospitals that rely on subsidies from insurance agents is important to inform policy direction. Finding of the study suggest the NHIS has had moderate improvement in the management of the hospitals studied. Managerial problems of the hospitals somewhat weigh in a balance with the benefits accruing to them. The successes of the hospitals under the scheme are seen as opportunities for growth. But the problems identified must be given priority by both the government and the NHIA to improve hospitals operation. In particular, logistical shortages, manual claims processing systems, waiting hours and moral hazard should be given critical attention. Efficiency gains should also form a major preoccupation of hospital managers to maximise financial security of the health facilities. Beyond these policy proposals, further research should be directed at the phenomena of moral hazard which creates sustainability problems for social health insurance schemes.

The findings albeit crucial for policy direction, it is important to acknowledge that a limitation of the study was the use of geographical accessibility to select districts. This approach lacked scientific merit though its application does not in any way invalidate the study’s findings. Since district hospitals in Ghana are quite heterogeneous, a variety of methodological approaches are required for future studies.

## Competing interest

SAM, FAB and FXA hold management positions in the NHIA. To avoid competing interest, they were distanced from the data collection. They also steered away from passing value judgement on the findings.

## Authors’ contribution

RAA originated the study and contributed to the design, data collection and preparation of the draft manuscript. SAM and FAB contributed to a review of relevant literature and shaped the methodology. FXA was instrumental in the presentation of results and discussion. All the Authors read and approved the final manuscript.

## Pre-publication history

The pre-publication history for this paper can be accessed here:

http://www.biomedcentral.com/1472-6963/12/174/prepub
